# Association of Autoimmune Diseases With Pancreatic Cancer: A Nationwide Follow‐Up Study From Sweden

**DOI:** 10.1002/cam4.71706

**Published:** 2026-03-07

**Authors:** Jiarong Gu, Yuhong Zhang, Kristina Sundquist, Jan Sundquist, Huifang Yang, Wenhua Ling, Xinjun Li

**Affiliations:** ^1^ School of Public Health Ningxia Medical University Yinchuan China; ^2^ Key Laboratory of Environmental Factors and Chronic Disease Control Ningxia Medical University Yinchuan China; ^3^ Department of Clinical Sciences, Center for Primary Health Care Research Lund University, and University Clinic Primary Care, Skåne University Hospital Malmö Sweden; ^4^ Department of Family and Community Medicine and of Epidemiology The University of Texas Health Science Center Houston Texas USA; ^5^ Department of Functional Pathology, Center for Community‐Based Healthcare Research and Education (CoHRE) School of Medicine, Shimane University Matsue Japan; ^6^ Guangdong Provincial Key Laboratory of Food, Nutrition and Health, and Department of Nutrition School of Public Health, SunYat‐sen University Guangzhou China

**Keywords:** autoimmune diseases, comorbidities, incidence ratios, mortality ratios, pancreatic cancer

## Abstract

**Background:**

The incidence of pancreatic cancer (PC) and autoimmune diseases (ADs) has been increasing worldwide. While certain associations between specific ADs, such as pancreatitis, and PC have long been well confirmed, population‐focused research investigating the broader spectrum of ADs and their relationship with PC risk remains limited.

**Methods:**

We implemented a cohort study based on population data to analyze the relationships between ADs and PC susceptibility. Diagnostic information on 43 ADs was obtained from the Swedish Inpatient Register (1964–2018), while cancer incidence and mortality data were sourced from the National Cancer Registry and Cause of Death Register starting in 1997. Relative cancer risks were quantified using standardized incidence ratios (SIRs) and standardized mortality ratios (SMRs).

**Results:**

Within the study's total population of 16.4 million, 1.1 million cases of ADs were identified, and 3257 patients were later diagnosed with PC accounting for 5.42% of all cancer cases in the cohort. The SIRs for PC in patients with ADs were 1.24 (men) and 1.19 (women); 12 ADs were positively correlated with the incidence of PC. The SMRs for PC in patients with ADs were 1.28 (men) and 1.22 (women); 15 ADs were positively correlated with PC mortality. When the follow‐up time was less than 1 year, the overall risk of PC in patients with ADs was 3.88; over 10 years, the risk reached 1.12.

**Conclusions:**

We have newly discovered the relationship between several ADs and the risk of PC incidence and mortality, including discoid lupus erythematosus, lupoid hepatitis, giant‐cell arteritis, and rheumatic fever. The results of this study back the notion that ADs may have a role in promoting the onset of PC.

## Graphical abstract

Graphic abstract illustrating the nationwide cohort design used to investigate the association between autoimmune diseases and the risk of pancreatic cancer in Sweden.

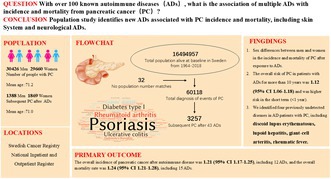

## 1 Introduction

The global incidence of pancreatic cancer (PC) is on the rise, with the highest mortality of any malignancy and a 5‐year overall survival of 5%–10% [1]. PC is characterized by a short disease course, rapid deterioration, short survival and poor prognosis [2]. One of the major reasons for this is that the diagnosis of PC often takes place when the disease is already advanced, and even with a wide range of therapeutic approaches, such as surgery, radiotherapy, biologic therapy, and targeted therapies, the overall survival of PC patients does not improve significantly [3, 4], thus limiting therapeutic choices and decreasing the prospect of a cure. Smoking, obesity, chronic pancreatitis, and diabetes are known traditional risk factors for PC [5]. It has been suggested that chronic inflammation and abnormal autoimmune surveillance are risk factors for certain types of cancer [6, 7].

Recent studies have uncovered notable similarities in the cellular landscapes of chronic inflammation and the tumor microenvironment when at the single‐cell level [8, 9]. Elucidating the genetic and biological mechanisms that underline PC in autoimmune diseases (ADs) could accordingly be pivotal to supplying guidance for the development of more efficient preventive and therapeutic strategies. ADs are a series of diseases caused by disorders of the immune system. There are hundreds of existing ADs involving multiple systems throughout the body, including the skin system, nervous system, and skeletal system. Current studies have shown that some ADs are positively associated with an increased risk of PC [10, 11]. For example, autoimmune pancreatitis (AIP) is highly correlated with PC; it may lead to an increased risk of cancer due to long‐term chronic inflammation [12]. The mechanism of type 1 diabetes involves the destruction of pancreatic β‐cells, and the incidence of type 1 diabetes is positively correlated with PC [13]. Patients with ADs taking immunosuppressive drugs lead to increased cancer risk.

In this study, leveraging data from a nationwide Swedish follow‐up study spanning 1964 to 2018, we conducted a large population‐based cohort research project focusing on the correlation between 43 ADs and PC, with the purpose of establishing the long‐term incidence and mortality of PC in patients with ADs.

## 2 Materials and Methods

### 2.1 Data Sources

We retrieved cancer data from the Swedish Cancer Registry and Cause of Death Register. The study obtained details on ADs from the National Inpatient Register (started in 1964) and the outpatient register (started in 2001), encompassing 43 different categories of ADs [14]. Information from these registers was linked to the population register at the individual level through the national 10‐digit civic registration number. With the merged dataset, serial numbers took the place of civic registration numbers to ensure the protection of people's integrity.

### 2.2 Identification of Cases and Selection of Control Subjects

PC patients were defined as participants with primary malignant disease. The incidence PC cases identified from the Cancer Register with the International Classification of Diseases version 7 (ICD‐7) code 157, the mortality due to PC was identified from the Death of Cause Register according to ICD‐7 to ICD‐9 codes 157, and ICD‐10 code C25.

### 2.3 Identification of Autoimmune Disorders and Other Exposures Factors

Patients with ADs were defined as those with at least one AD. The first‐diagnosed ADs were served as the focus of the basic analysis. The ICD codes employed to identify ADs are provided in Table S1.

### 2.4 Covariates

The Total Population Register provided data on individual‐level sociodemographic characteristics. Age and time period were divided into 5‐years. Educational attainment was grouped into three tiers: < 9 years (compulsory school or less), 10–11 years (practical high school or some theoretical high school), and ≥ 12 years (theoretical high school and/or college). Country of origin was categorized as “born in Sweden” and “born outside Sweden.” Region of residence was categorized into “small towns/rural areas,” “middle‐sized towns,” or “large cities” (Stockholm, Gothenburg, and Malmö). During the study period, comorbidities were retrieved from the National Patient Register over the study period and defined as follows: obesity (ICD‐7: 287.00, 287.09, ICD‐8: 277.99, ICD‐9: 278.0; ICD‐10 E65–E68); chronic obstructive pulmonary disease (ICD‐7 500–502, ICD‐8: 490–493, ICD‐9 490–496, ICD‐10 J40–J49); and alcoholism (ICD‐7: 307, 322, 581, ICD‐8: 291, 303, 571, ICD‐9: 291, 303, 571, ICD‐10: F10 and K70).

### 2.5 Statistical Analysis

The calculation of person‐year commenced from the initial diagnosis of AD in 1964 and continued until one of the following endpoints was reached: PC diagnosis, death, emigration, or the conclusion of the follow‐up in 2018. Standardized incidence ratios (SIRs) and standardized mortality ratios (SMRs) were adjusted for several factors: sex, age (divided into 5‐year categories for patients of all ages), time (divided into 5‐year periods to adjust for possible variations in rates of hospital admission), and comorbidities. To address both the long developmental timeline of PC and surveillance bias arising from the ADs, we further conducted sensitivity analyses. These analyses excluded patients with preexisting AIP, aiming to explore the associations of ADs with all primary PCs. All the statistical tests adopted two‐sided approach, and statistical significance was defined as *p* value < 0.05. SAS version 9.4 was used to conduct the analyses.

## 3 Results

Table S1 shows different versions of the ICDs and the number of ADs. The three most frequently occurring ADs included psoriasis (174,025, 16.6%), rheumatoid arthritis (130,687, 12.5%), and type I diabetes (106,615, 10.2%).

The characteristics of the study population, together with the number of patients with PC and ADs, can be found in Table 1 and Table S2. The study population included 16.4 million individuals followed from 1964 to 2018, during which 60,086 PCs were diagnosed at a mean age of 71.2 years. The world age‐standardized incidence rates per 100,000 person‐years were 6.56 (95% CI 6.60–6.61) overall, 7.37 (95% CI 7.29–7.46) for men and 5.83 (95% CI 5.77–5.90) for women. Of all PC cases, 3257 (5.42%) had a previous diagnosis of an AD. Table 1 also displays PC incidence rates stratified by sex. Men were diagnosed with PC at a mean age of 69.5 years, while the mean diagnostic age for women was 72.2 years. An incidence rate for PC after ADs of 4.54/100,000 person years was observed among men and 3.57/100,000 person years among women.

**TABLE 1:** Characteristics of study population and number of cases of pancreatic cancer, 1964–2018.

	Men	Women	All
Total population	8,382,433	8,112,524	16,494,657
Total events	30,426	29,660	60,086
Age range	7–102	15–103	7–103
Mean age at diagnosis (years) ± SD	69.9 ± 10.6	72.1 ± 10.9	71.2 ± 10.8
Incidence rate per 100,000 person years (95% CI)^a^	7.37 (7.29, 7.46)	5.83 (5.77, 5.90)	6.56 (6.60, 6.61)
Subsequent events after autoimmune diseases			
Total events	1388	1869	3257
Age range	13–96	19–100	13–100
Mean age at diagnosis (years) ± SD	69.5 ± 10.5	72.2 ± 10.1	71.0 ± 10.4
Incidence rate per 100,000 person years (95% CI)^a^	4.54 (4.48, 4.59)	3.57 (3.53, 3.62)	3.95 (3.92, 3.99)

^a^ Adjusted for world standardized population.

The risk of PC developing after an AD is shown in Table 2, focusing only on ADs with a minimum of 10 cases or any significant associations. The overall PC risk following hospitalization for an AD was 1.21 (95% CI 1.17–1.25), and for 12 out of the 43 studied ADs, hospitalization was linked to a higher SIR. The highest SIRs were polymyositis (PM) (1.89), celiac disease (1.84), and discoid lupus erythematosus (DLE) (1.82). There were several differences between men and women: the 95% CI for men was 1.24 (95% CI 1.18–1.31), and that for women was 1.19 (95% CI 1.14–1.25). The male‐specific ADs were grave disease (1.37), rheumatic fever (1.64), and ulcerative colitis (1.33), and the female‐specific ADs were PM (2.14), systemic lupus erythematosus (SLE) (1.84), and psoriasis (1.50).

**TABLE 2:** Subsequent incidence risk of pancreas cancer after autoimmune diseases, 1964–2018.

Autoimmune condition	Men				Women				All			
	O	SIR	95% CI		O	SIR	95% CI		O	SIR	95% CI	
Addison disease	4	1.32	0.34	3.42	6	1.26	0.45	2.77	10	1.29	0.61	2.38
Ankylosing spondylitis	34	1.11	0.77	1.55	12	1.00	0.51	1.75	46	1.08	0.79	1.44
Celiac disease	33	**1.93**	**1.33**	**2.72**	45	**1.77**	**1.29**	**2.37**	78	**1.84**	**1.45**	**2.29**
Chronic rheumatic heart disease	46	0.96	0.70	1.29	50	0.97	0.72	1.28	96	0.97	0.78	1.18
Crohn disease	56	1.26	0.95	1.63	59	1.25	0.95	1.61	115	**1.25**	**1.03**	**1.50**
Dermatitis Herpetiformis	8	1.22	0.52	2.41	6	1.46	0.53	3.20	14	1.31	0.71	2.20
Diabetes mellitus type I	283	**1.77**	**1.57**	**1.99**	172	**1.61**	**1.38**	**1.87**	455	**1.71**	**1.56**	**1.87**
Discoid lupus erythematosus	7	2.17	0.86	4.50	13	1.67	0.88	2.86	20	**1.82**	**1.11**	**2.81**
Giant‐cell arteritis	18	0.98	0.58	1.55	38	1.02	0.72	1.40	56	1.00	0.76	1.30
Glomerular nephritis chronic	26	0.87	0.57	1.27	14	0.92	0.50	1.55	40	0.89	0.63	1.21
Glomerular nephritis acute	8	0.66	0.28	1.31	4	0.55	0.14	1.42	12	0.62	0.32	1.09
Grave disease	52	**1.37**	**1.03**	**1.80**	185	**1.16**	**1.00**	**1.34**	237	**1.20**	**1.05**	**1.37**
Guillain‐Barre Syndrome	12	1.22	0.63	2.15	4	0.60	0.16	1.55	16	0.97	0.55	1.58
Hashimoto thyroiditis	26	1.43	0.93	2.10	78	0.99	0.78	1.24	104	1.07	0.88	1.30
Immune thrombocytopenic purpura	20	1.63	0.99	2.52	20	1.30	0.79	2.02	40	**1.45**	**1.03**	**1.98**
Lupoid hepatitis	17	1.60	0.93	2.57	22	1.46	0.92	2.22	39	**1.52**	**1.08**	**2.08**
Multiple sclerosis	17	0.66	0.38	1.05	36	0.79	0.55	1.09	53	0.74	0.55	0.97
Myasthenia gravis	3	0.35	0.07	1.05	11	1.43	0.71	2.57	14	0.87	0.47	1.46
Pemphigoid	13	1.48	0.79	2.54	12	1.23	0.63	2.15	25	1.35	0.87	1.99
Pernicious anemia	55	**1.70**	**1.28**	**2.22**	56	**1.57**	**1.19**	**2.04**	111	**1.63**	**1.34**	**1.97**
Polymyalgia rheumatica	63	0.99	0.76	1.27	142	1.16	0.98	1.37	205	1.10	0.96	1.26
Polymyositis/dermatomyositis	6	1.56	0.56	3.41	11	**2.14**	**1.06**	**3.85**	17	**1.89**	**1.10**	**3.04**
Primary biliary cirrhosis	2	0.71	0.07	2.61	12	1.03	0.53	1.80	14	0.97	0.53	1.62
Psoriasis	191	1.15	0.99	1.32	264	**1.50**	**1.32**	**1.69**	455	**1.33**	**1.21**	**1.45**
Rheumatic fever	25	**1.64**	**1.06**	**2.42**	6	1.01	0.36	2.22	31	1.46	0.99	2.08
Rheumatoid arthritis	140	0.90	0.76	1.06	349	1.00	0.90	1.11	489	0.97	0.89	1.06
Sarcoidosis	47	1.33	0.98	1.77	43	1.04	0.75	1.41	90	1.17	0.94	1.44
Sjögren's syndrome	3	0.92	0.17	2.72	36	1.24	0.87	1.72	39	1.21	0.86	1.65
Systemic lupus erythematosus	10	1.67	0.80	3.08	42	**1.84**	**1.33**	**2.49**	52	**1.80**	**1.35**	**2.37**
Systemic sclerosis	6	1.94	0.70	4.24	10	1.14	0.54	2.11	16	1.35	0.77	2.20
Ulcerative colitis	127	**1.33**	**1.11**	**1.58**	94	1.21	0.98	1.48	221	**1.27**	**1.11**	**1.45**
All	1388	**1.24**	**1.18**	**1.31**	1869	**1.19**	**1.14**	**1.25**	3257	**1.21**	**1.17**	**1.25**

*Note:* Autoimmune diseases with fewer than 10 cases were excluded but included in total numbers. Bold type: 95% confidence interval does not include 1.00.

Abbreviations: CI, confidence intervals; O, observed cases; SIR, standardized incidence ratio.

Table 3 presents the association between ADs follow‐up duration and PC risk. The overall risk (3.88) was observed during follow‐up of less than 1 year—this period also saw the lowest number of diagnosed cancers. As follow‐up duration lengthened, the number of cancers increased; at follow‐up periods exceeding 10 years, the risk dropped to 1.12 (95% CI 1.06–1.18). Short‐term risk (less than 1 year) included 14 ADs, and long‐term risk (more than 10 years) included 7 ADs. Interestingly, diabetes type I (13.68) and celiac disease (14.19) had the highest short‐term risk of PC, but both had a decreased risk in the long term. Among individuals with ADs, the SIR for PC was slightly higher in men than in women. As detailed in Tables S3 and S4, the overall PC risk after AD‐related hospitalization was 1.15 (95% CI 1.05–1.25) for males and 1.10 (95% CI 1.02–1.18) for females, respectively.

**TABLE 3:** Subsequent risk of pancreas cancer after autoimmune diseases according to the length of follow‐up time, 1964–2018.

Autoimmune condition	Follow‐up (years)															
	< 1				1–4				5–9				≥ 10			
	O	SIR	95% CI		O	SIR	95% CI		O	SIR	95% CI		O	SIR	95% CI	
Addison disease	1	3.03	0.00	17.37	4	2.05	0.53	5.30	1	0.53	0.00	3.02	4	1.11	0.29	2.87
Ankylosing spondylitis	1	1.02	0.00	5.85	9	1.25	0.57	2.38	5	0.61	0.19	1.44	31	1.18	0.80	1.67
Celiac disease	21	**14.19**	**8.77**	**21.73**	25	**2.27**	**1.47**	**3.35**	14	1.16	0.63	1.95	18	1.00	0.59	1.59
Chronic rheumatic heart disease	6	1.07	0.39	2.35	22	0.78	0.49	1.19	21	0.84	0.52	1.29	47	1.15	0.85	1.53
Crohn disease	12	**5.29**	**2.72**	**9.26**	27	**1.73**	**1.14**	**2.52**	22	1.29	0.80	1.95	54	0.95	0.71	1.24
Dermatitis Herpetiformis	1	2.94	0.00	16.86	4	1.56	0.41	4.04	3	1.09	0.21	3.24	6	1.19	0.43	2.60
Diabetes mellitus type I	187	**13.68**	**11.79**	**15.79**	115	**1.21**	**1.00**	**1.45**	83	0.96	0.76	1.19	70	0.99	0.77	1.25
Discoid lupus erythematosus	2	4.76	0.45	17.51	5	1.61	0.51	3.78	3	0.95	0.18	2.81	10	**2.32**	**1.10**	**4.28**
Giant‐cell arteritis	5	1.28	0.40	3.02	26	1.08	0.71	1.59	11	0.60	0.30	1.08	14	1.45	0.79	2.45
Glomerular nephritis chronic	5	1.93	0.61	4.54	10	0.91	0.43	1.68	9	0.92	0.42	1.75	16	0.74	0.42	1.20
Glomerular nephritis acute	2	4.26	0.40	15.65	0				1	0.37	0.00	2.12	9	0.66	0.30	1.26
Grave disease	18	**3.10**	**1.84**	**4.91**	40	0.98	0.70	1.33	55	1.31	0.99	1.71	124	1.14	0.95	1.37
Guillain‐Barre Syndrome	2	3.51	0.33	12.90	4	1.06	0.27	2.73	3	0.77	0.14	2.27	7	0.86	0.34	1.77
Hashimoto thyroiditis	15	**3.04**	**1.69**	**5.02**	35	1.12	0.78	1.56	21	0.80	0.49	1.22	33	0.95	0.66	1.34
Immune thrombocytopenic purpura	5	**3.40**	**1.07**	**8.00**	11	1.32	0.65	2.37	2	0.30	0.03	1.11	22	**1.97**	**1.23**	**2.98**
Lupoid hepatitis	7	**8.64**	**3.43**	**17.91**	7	1.46	0.58	3.03	5	1.04	0.33	2.46	20	1.31	0.80	2.03
Multiple sclerosis	5	2.86	0.90	6.72	7	0.54	0.21	1.11	9	0.55	0.25	1.05	32	0.79	0.54	1.12
Myasthenia gravis	1	1.37	0.00	7.85	3	0.64	0.12	1.90	2	0.50	0.05	1.83	8	1.19	0.51	2.36
Pemphigoid	4	1.78	0.46	4.60	13	1.39	0.74	2.39	5	1.11	0.35	2.61	3	1.22	0.23	3.61
Pernicious anemia	15	**3.90**	**2.17**	**6.44**	34	**1.45**	**1.00**	**2.02**	29	**1.54**	**1.03**	**2.22**	33	**1.51**	**1.04**	**2.13**
Polymyalgia rheumatica	28	**2.58**	**1.71**	**3.73**	79	1.15	0.91	1.43	50	0.90	0.67	1.19	48	0.95	0.70	1.25
Polymyositis/dermatomyositis	3	**6.12**	**1.15**	**18.12**	6	2.21	0.80	4.85	4	1.69	0.44	4.38	4	1.17	0.30	3.02
Primary biliary cirrhosis	3	3.23	0.61	9.55	4	0.98	0.26	2.54	0				7	1.24	0.49	2.57
Psoriasis	26	**1.83**	**1.20**	**2.69**	124	1.17	0.97	1.39	136	**1.31**	**1.10**	**1.55**	169	**1.42**	**1.22**	**1.65**
Rheumatic fever	0				9	**3.38**	**1.53**	**6.45**	3	1.00	0.19	2.96	19	1.26	0.76	1.97
Rheumatoid arthritis	33	**1.65**	**1.14**	**2.33**	125	0.90	0.75	1.07	127	0.94	0.78	1.12	204	0.97	0.84	1.11
Sarcoidosis	13	**6.57**	**3.48**	**11.26**	10	0.73	0.35	1.34	23	1.54	0.98	2.32	44	0.96	0.69	1.28
Sjögren syndrome	1	0.65	0.00	3.72	9	0.82	0.37	1.56	21	**2.08**	**1.29**	**3.18**	8	0.83	0.35	1.63
Systemic lupus erythematosus	3	2.94	0.55	8.71	15	**2.26**	**1.26**	**3.74**	7	1.04	0.41	2.15	27	**1.87**	**1.23**	**2.73**
Systemic sclerosis	1	1.89	0.00	10.82	3	0.93	0.18	2.77	1	0.32	0.00	1.86	11	**2.19**	**1.09**	**3.93**
Ulcerative colitis	13	**2.73**	**1.45**	**4.68**	32	0.94	0.64	1.33	44	1.18	0.85	1.58	132	**1.36**	**1.14**	**1.61**
All	449	**3.88**	**3.53**	**4.25**	828	**1.10**	**1.03**	**1.18**	732	1.04	0.97	1.12	1248	**1.12**	**1.06**	**1.18**

*Note:* Bold type: 95% confidence interval does not include 1.00.

Abbreviations: CI, confidence intervals; O, observed cases; SIR, standardized incidence ratio.

Finally, we employed SMRs to assess the mortality risk of AD patients and those with PC. Table 4 shows that a total of 4702 deaths were observed, with a mortality risk of 1.24 (95% CI 1.21–1.28), of which higher risk factors for death were type I diabetes (2.04, 95% CI 1.90–2.19) and SLE (1.79, 95% CI 1.39–2.27). The SMRs for PCs was marginally higher in men with ADs compared to women with ADs.

**TABLE 4:** Subsequent mortality risk of pancreas cancer after comorbidities, 1964–2018.

Autoimmune condition	Men				Women				All			
	O	SMR	95% CI		O	SMR	95% CI		O	SMR	95% CI	
Addison disease	6	1.43	0.51	3.12	8	1.16	0.50	2.30	14	1.26	0.69	2.12
Amyotrophic lateral sclerosis	14	1.17	0.64	1.96	5	0.73	0.23	1.72	19	1.01	0.61	1.58
Ankylosing spondylitis	40	1.02	0.72	1.38	16	1.03	0.59	1.68	56	1.02	0.77	1.32
Celiac disease	39	**1.65**	**1.17**	**2.26**	64	**1.80**	**1.39**	**2.30**	103	**1.74**	**1.42**	**2.11**
Chronic rheumatic heart disease	52	0.82	0.61	1.08	69	0.95	0.74	1.20	121	0.89	0.74	1.07
Crohn disease	70	1.19	0.93	1.50	86	**1.33**	**1.06**	**1.64**	156	**1.26**	**1.07**	**1.48**
Dermatitis Herpetiformis	10	1.05	0.50	1.94	6	0.99	0.36	2.17	16	1.03	0.59	1.67
Diabetes mellitus type I	443	**2.06**	**1.87**	**2.26**	322	**2.02**	**1.81**	**2.25**	765	**2.04**	**1.90**	**2.19**
Discoid lupus erythematosus	7	1.58	0.63	3.28	14	1.23	0.67	2.08	21	1.33	0.82	2.04
Giant‐cell arteritis	35	1.14	0.79	1.59	85	**1.25**	**1.00**	**1.54**	120	**1.21**	**1.01**	**1.45**
Glomerular nephritis chronic	34	0.90	0.62	1.26	14	0.72	0.39	1.20	48	0.84	0.62	1.11
Glomerular nephritis acute	12	0.76	0.39	1.34	8	0.79	0.34	1.56	20	0.77	0.47	1.20
Grave disease	69	**1.37**	**1.06**	**1.73**	266	**1.21**	**1.07**	**1.36**	335	**1.24**	**1.11**	**1.38**
Guillain‐Barre Syndrome	13	0.97	0.51	1.66	8	0.83	0.35	1.64	21	0.91	0.56	1.39
Hashimoto thyroiditis	36	1.42	0.99	1.97	119	1.05	0.87	1.26	155	1.12	0.95	1.31
Immune thrombocytopenic purpura	30	**1.72**	**1.16**	**2.45**	28	1.26	0.84	1.83	58	**1.46**	**1.11**	**1.89**
Lupoid hepatitis	18	1.31	0.78	2.08	31	**1.49**	**1.01**	**2.12**	49	**1.42**	**1.05**	**1.88**
Multiple sclerosis	24	0.75	0.48	1.11	53	0.92	0.69	1.21	77	0.86	0.68	1.07
Myasthenia gravis	13	1.04	0.55	1.78	14	1.21	0.66	2.04	27	1.12	0.74	1.63
Pemphigoid	19	1.27	0.76	1.98	19	1.01	0.61	1.59	38	1.13	0.80	1.55
Pernicious anemia	67	**1.55**	**1.20**	**1.97**	73	**1.45**	**1.14**	**1.83**	140	**1.50**	**1.26**	**1.77**
Polymyalgia rheumatica	98	1.00	0.81	1.22	229	1.12	0.98	1.27	327	1.08	0.96	1.20
Polymyositis/dermatomyositis	6	1.14	0.41	2.50	16	**2.19**	**1.25**	**3.56**	22	**1.75**	**1.10**	**2.65**
Primary biliary cirrhosis	2	0.52	0.05	1.93	24	1.46	0.93	2.17	26	1.28	0.84	1.88
Psoriasis	277	**1.25**	**1.10**	**1.40**	300	**1.21**	**1.08**	**1.35**	577	**1.23**	**1.13**	**1.33**
Reiter disease	10	1.34	0.64	2.48	0				10	1.18	0.56	2.18
Rheumatic fever	30	**1.54**	**1.04**	**2.20**	10	1.23	0.59	2.28	40	**1.45**	**1.03**	**1.97**
Rheumatoid arthritis	196	0.92	0.80	1.06	524	1.07	0.98	1.16	720	1.02	0.95	1.10
Sarcoidosis	54	1.19	0.89	1.55	61	1.05	0.80	1.35	115	1.11	0.92	1.33
Sjögren syndrome	5	1.02	0.32	2.39	46	1.10	0.80	1.46	51	1.09	0.81	1.43
Systemic lupus erythematosus	13	1.65	0.88	2.83	55	**1.83**	**1.38**	**2.38**	68	**1.79**	**1.39**	**2.27**
Systemic sclerosis	8	1.94	0.83	3.84	17	1.43	0.83	2.29	25	**1.56**	**1.01**	**2.31**
Ulcerative colitis	169	**1.32**	**1.13**	**1.53**	142	**1.30**	**1.10**	**1.53**	311	**1.31**	**1.17**	**1.46**
All	1946	**1.28**	**1.23**	**1.34**	**2756**	**1.22**	**1.17**	**1.27**	4702	**1.24**	**1.21**	**1.28**

*Note:* Autoimmune diseases with fewer than 10 cases were excluded but included in total numbers. Bold type: 95% confidence interval does not include 1.00.

Abbreviations: CI, confidence intervals; O, observed cases; SMR, standardized mortality ratio.

After excluding those with AIP, we performed a sensitivity analysis, and the short‐term risk of death (< 1 year) was 2.24 (95% CI 2.20–2.29), the long‐term risk of death (more than 10 years) was 1.16 (95% CI 1.11–1.21), and diabetes mellitus type I (8.11, 95% CI 6.91–9.45) and celiac disease (5.58, 95% CI 2.77–10.03) were the most prominent risk factors for short‐term mortality in patients with PC, as detailed in Table S5.

## 4 Discussion

This study, conducted on a large Swedish population and encompassing 43 ADs, represents the most extensive investigation to date on the relationship between ADs and PC. The population‐based prevalence of PC was 6.56%. The overall incidence risk of developing PC following an AD diagnosis was 1.21, with 12 ADs identified as being associated with an increased risk of PC. Additionally, the risk of death from PC was 1.24, and 15 ADs were linked to higher PC‐related mortality. Notably, sex‐specific differences were observed, with seven immune disorders remaining significantly associated with PC even 10 years after the initial AD diagnosis. These findings align with previous research conducted in the US, which identified 8 ADs associated with PC incidence [15].

The incidence and prevalence of several ADs are higher in women than in men, such as SLE, rheumatoid arthritis, and inflammatory bowel disease. However, our study revealed that the total number of women with ADs was lower than that of men in the study population. This observation may be because our study was only based on hospital‐ and specialist clinic data and it is possible that some of the female AD cases in the population were diagnosed at primary health care clinics; such data were not used in the present study. However, our aim was to assess the association between AD and PC rather than the incidence of ADs in the population.

Notably, the incidence rate of PC was 3.88 within the first year following diagnosis of ADs, which was significantly higher than that observed in the subsequent 10‐year period. For patients in Japan, SIR for cancer development within the first year after a diagnosis of AIP reached 6.1, markedly exceeding the SIR of 1.5 in the years thereafter [16]. Additionally, the SIR of cancer among patients with IgG4‐related disease or AIP ranged from 2.7 to 3.8 [17]. Our findings are consistent with these observations. First, when AD is diagnosed due to overt clinical symptoms, PC may already be in its early stage, as the onset and progression of PC often preceded the manifestation of noticeable autoimmune symptoms. Second, following an AD diagnosis, patients typically undergo more frequent medical surveillance (regular imaging scans and laboratory tests for disease monitoring), and this enhanced surveillance can significantly improve the detection rate of early‐stage PC. Third, paraneoplastic syndrome also warrants consideration: early occult PC may activate the immune system through molecular mimicry, thereby inducing autoimmune symptoms; subsequent workup for these autoimmune manifestations may then lead to the identification of the underlying tumor [18].

The association between AIP and PC has been studied before [19]. However, several studies have demonstrated that the cancer risk in patients with AIP—both before and after the diagnosis of AIP—is comparable to that in control populations, with no significant increase in cancer risk observed in either period [20, 21]. A potential explanation for this discrepancy may lie in the diagnostic approach employed in our study, which relied heavily on imaging and histopathological findings but lacked integration of serological markers (IgG4) or assessment of response to steroid therapy [22]. Additionally, our study focused specifically on type 1 AIP. Given that type 2 AIP is a rare subtype and is generally considered a benign condition [23], larger‐scale studies are warranted in the future to elaborate on the relationship between AIP subtypes and cancer risk.

The elevated risk of PC associated with other autoimmune disorders—such as type 1 diabetes mellitus, Crohn's disease, ulcerative colitis, PM, DLE, and coeliac disease—remains attention in subsequent academic investigations and clinical practices. Patients with PM exhibit a significantly elevated risk of developing PC, with a SIR ranging from 2.0 to 3.0; notably, this risk peaks within 1–3 years following PM diagnosis [24]. A large US cohort study (*n* > 150,000) demonstrated that patients with celiac disease (CD) have a 29% increased risk of PC, with the highest risk observed in the first year after CD diagnosis (hazard ratio [HR] = 1.56) [25]. While studies directly investigating the association between DLE and PC remain limited, patients with SLE—a disease with which DLE is either associated as a subtype or linked as an independent factor—show a 42% increased risk of PC, suggesting that DLE may confer a similar risk [26]. These findings are consistent with those of our study, indicating that ADs are associated with an increased risk of PC, albeit with distinct underlying mechanisms specific to each AD subtype. Specifically, PM is characterized by chronic inflammation of skeletal muscle, accompanied by the systemic release of proinflammatory cytokines (e.g., TNF‐α, IL‐6, IL‐1β). These cytokines may diffuse to pancreatic tissues via systemic circulation, thereby promoting pancreatic tumor cell proliferation, angiogenesis, and immune evasion [27]. In patients with CD, disruption of the intestinal mucosal barrier may lead to an increased production of anti‐tissue transglutaminase (tTG) antibodies, which may target pancreatic acinar cells and induce AIP‐like lesions, ultimately progressing to PC [28, 29]. For patients with DLE, plasma levels of interferon‐α (IFN‐α) are significantly elevated; IFN‐α activates the JAK–STAT signaling pathway to promote pancreatic tumor cell proliferation, inhibit apoptosis, and recruit myeloid‐derived suppressor cells, thereby establishing an immunosuppressive microenvironment [30]. Additionally, DLE frequently coexists with SLE, a phenomenon that may be associated with excessive B‐cell activation and dysregulated immune modulation [31, 32]. In conclusion, these ADs synergistically increase the risk of PC through inflammatory mechanisms and disease‐specific pathways. Early intervention targeting immune dysregulation may inhibit this pathogenic process.

ADs may promote PC through various mechanisms, including chronic inflammation, immune regulation abnormalities, and genetic alterations [33, 34]. Chronic inflammation is a key shared characteristic of ADs and PC. During inflammation, immune cells such as macrophages and neutrophils release reactive oxygen species (ROS) and reactive nitrogen species (RNS), which cause DNA damage and promote mutation accumulation in pancreatic cells [35]. Additionally, cytokines like TNF‐α and IL‐6, released by the inflammatory system, activate the NF‐κB and STAT3 inflammatory signaling pathways, further contributing to PC development [36]. Moreover, the long‐term treatment of ADs, often required to manage these chronic conditions, may inadvertently increase cancer risk [37]. Our study has also identified new associations between PC and ADs, including DLE, lupoid hepatitis, giant‐cell arteritis, and rheumatic fever. Further observational and animal studies are needed to validate these findings and to uncover the underlying mechanisms linking ADs to PC.

The relationship between pernicious anemia and PC remains controversial. An earlier study based on a Swedish population reported a SIR of 1.7 for PC in patients with pernicious anemia [38]. Similarly, a case–control study using the SEER‐Medicare population in the US [39]. Identified a significant positive correlation between pernicious anemia and PC risk, aligning with our findings. However, other studies have reported conflicting results. For instance, a US cohort study and a UK case–control study [40] found no association between pernicious anemia and PC. These inconsistencies highlight the need for future studies with larger sample sizes and robust methodologies to clarify the relationship between pernicious anemia and PC.

The strengths of our study include the large number of PC patients with rare ADs. The Swedish Cancer Registry, which systematically records all cases, minimizes recall bias by integrating data from the national healthcare system. Additionally, the chronological documentation of disease onset reduces the likelihood of reverse causation, where preexisting cancer might contribute to the development of autoimmunity [41].

Our study also has several limitations. The low incidence of rare ADs resulted in a small sample size of PC patients. Additionally, many ADs were first identified using ICD‐10 diagnostic codes, which may affect data accuracy. Longer follow‐up periods are needed to analyze temporal trends more effectively and clarify the relationship between ADs and PC risk.

In conclusion, 5.42% of PC patients had a prior diagnosis of an AD, with an overall risk ratio of 1.24. Further population‐based and experimental studies are needed to validate our findings and clarify the mechanisms through which immune dysregulation contributes to the development of PC. Pancreatic cancer has a poor prognosis; the risk of PC due to immune dysregulation can be reduced by effective management and treatment of ADs, including suppression of excessive immune responses and chronic inflammation. Interventions targeting modifiable risk factors for PC, such as smoking and alcohol restriction, which in pancreas‐associated ADs may further increase cancer risk [42]. Exploring global cancer risk would help clarify whether the observed association with PC is part of a broader predisposition to malignancy or is specific to pancreatic tissue—an issue that is highly relevant to understanding the potential underlying mechanism.

## Author Contributions

**Jiarong Gu:** conceptualization (equal), writing – original draft (equal). **Yuhong Zhang:** writing – review and editing (equal). **Kristina Sundquist:** data curation (equal), resources (equal), writing – review and editing (equal). **Jan Sundquist:** data curation (equal), resources (equal). **Huifang Yang:** writing – review and editing (equal). **Wenhua Ling:** conceptualization (equal), writing – review and editing (equal). **Xinjun Li:** conceptualization (equal), validation (equal).

## Funding

Financial support for this work was provided via grants to Kristina Sundquist: the Swedish Research Council and ALF funding from Region Skåne. The funders had no role in any aspect of the study, including study design and conduct, data handling (collection, management, analysis, interpretation), manuscript preparation, review, approval, or the decision to submit the manuscript for publication.

## Ethics Statement

This research received ethical approval from the Regional Ethical Review Board affiliated with Lund University in Sweden. All procedures throughout the study adhered to the principles outlined in the Helsinki Declaration, as well as the specific guidelines approved by the review board. Notably, an explicit note was made that the acquisition of informed consent was not required for this study. In terms of its nature, this is a nationwide register‐based research project, which utilizes pseudonymous for analysis.

## Conflicts of Interest

The authors declare no conflicts of interest.

## Supporting information


**Table S1:** ICD codes of autoimmune disorders, 1964–2018.
**Table S2:** Study population and number of events of pancreas cancer, 1964–2018.
**Table S3:** Subsequent incidence risks of pancreas cancer of patients with autoimmune disease in men, 1964–2018.
**Table S4:** Subsequent incidence risks of pancreas cancer of patients with autoimmune disease in women, 1964–2018.
**Table S5:** Subsequent mortality risk of pancreas cancer without autoimmune pancreatitis, 1964–2018.

## Data Availability

Several national registers were used in this study. Owing to ethical constraints, the data is not openly accessible, and we are not permitted to share it. Further information on the health registries is available from two sources: the Swedish National Board of Health and Welfare (website: https://www.socialstyrelsen.se/en/statistics‐and‐data/registers/) and co‐author Kristina Sundquist, who possesses the ethical permission for this study.
