# Supplementary material for: Association of Autoimmune Diseases With Pancreatic Cancer: A Nationwide Follow‐Up Study From Sweden
Source: Cancer Med. 2026 Mar 7;15(3):e71706. doi: 10.1002/cam4.71706 (PMC12967486; doi:10.1002/cam4.71706)
Supplement: Supplementary file 1 — Table S1: ICD codes of autoimmune disorders, 1964–2018. Table S2: Study population and number of events of pancreas cancer, 1964–2018. Table S3: Subsequent incidence risks of pancreas cancer of patients with autoimmune disease in men, 1964–2018. Table S4: Subsequent incidence risks of pancreas cancer of patients with autoimmune disease in women, 1964–2018. Table S5: Subsequent mortality risk of pancreas cancer without autoimmune pancreatitis, 1964–2018. [file CAM4-15-e71706-s001.docx]

**Supplementary materials:**

**Supplementary Table S1.** ICD codes of autoimmune disorders, 1964-2018.

**Supplementary Table S2.** Study population and number of events of pancreas cancer, 1964-2018.

**Supplementary Table S3.** Subsequent incidence risks of pancreas cancer of patients with autoimmune disease in men, 1964-2018.

**Supplementary Table S4.** Subsequent incidence risks of pancreas cancer of patients with autoimmune disease in women, 1964-2018.

**Supplementary Table S5.** Subsequent mortality risk of pancreas cancer without autoimmune pancreatitis, 1964-2018.

| **Supplementary Table S1. ICD codes of autoimmune disorders, 1964-2018.** | | | | | | |
| --- | --- | --- | --- | --- | --- | --- |
|  | ICD code | | | | No. of events | |
| Autoimmune condition | ICD-7 (1964-1968) | ICD-8 (1969-1986) | ICD-9 (1987-1996) | ICD-10 (1997-) | No. | % |
| Addison’s disease | 274.4 | 255.1 | 255E | E27.1, E27.2 | 3610 | 0.3 |
| Amyotrophic lateral sclerosis | 356.1 | 348 | 335C | G12.2 | 11815 | 1.1 |
| Angiitis hypersensitiva | none | 446.1 | 446C | M31.0 | 391 | 0.0 |
| Ankylosing spondylitis | 722.1 | 712.4 | 720A | M45, M08.1 | 19772 | 1.9 |
| Autoimmune hemolytic anemia | 292.10,292.20 | 283.90, 283.91 | 283A | D59.0, D59.1 | 1472 | 0.1 |
| Behçet’s disease | 138.14 | 136.07 | 136B | M35.2 | 1078 | 0.1 |
| Celiac disease | 286.00 | 269.00, 269.98 | 579A | K90.0 | 52842 | 5.0 |
| Chorea minor | 402 | 392.9 | 392 | I02 | 142 | 0.0 |
| Chronic rheumatic heart disease | 410-416 | 393-398 | 393-398 | I05-I09 | 24997 | 2.4 |
| Crohn’s disease | 572.00, 572.09 | 563.00 | 555 | K50 | 45678 | 4.3 |
| Dermatitis Herpetiformis | 704.00 | 693.99 | 694A | L13.0 | 2750 | 0.3 |
| Diabetes mellitus type I | 260 (age < 20 years) | 250 (age < 20 years) | 250 (age < 20 years) | E10 | 106529 | 10.1 |
| Discoid lupus erythematosus | 705.4 | 695.4 | 695E | L93.0 | 4283 | 0.4 |
| Giant-cell arteritis | none | 446.4 | 446F | M31.5, M31.6 | 23341 | 2.2 |
| Glomerular nephritis acute | 590 | 580 | 580 | N00, N01 | 18912 | 1.8 |
| Glomerular nephritis chronic | 592 | 582 | 582 | N03 | 8959 | 0.8 |
| Graves’ disease | 252 | 242 (not 242.1) | 242A, D, X | E05.0, E05.5 | 68157 | 6.5 |
| Guillain-Barré syndrome | 364.2 | 354.01 | 357A | G61.0 | 5090 | 0.5 |
| Hashimoto’s thyroiditis | 254.0,253.19,253.29 | 245.03, 244.09 | 245C, 244W, X | E03.5, E03.8, E03.9, E06.3 | 61507 | 5.8 |
| Immune thrombocytopenic purpura | 296.03 | 287.1 | 287D | D69.3 | 14293 | 1.4 |
| Localized scleroderma | 710.07 | 701.0 | 701A | L94.0 | 3847 | 0.4 |
| Lupoid hepatitis | 583.29 | 573.9 | 571E | K75.4 | 7492 | 0.7 |
| Multiple sclerosis | 345 | 340 | 340 | G35 | 31894 | 3.0 |
| Myasthenia gravis | 744.0 | 733.0 | 358A | G70.0 | 5125 | 0.5 |
| Pemphigoid | 704.01 | 694.02 | 694F,694G | L12 (not L12.2) | 9327 | 0.9 |
| Pemphigus | 704.10,704.11,704.19 | 694.00,694.01,694.09 | 694E | L10 (not L10.3 and L10.5) | 1775 | 0.2 |
| Pernicious anemia | 290.0,290.1 | 281 | 281A | D51.0 | 12282 | 1.2 |
| Polyarteritis nodosa | 456.1 | 446.0 | 446A | M30.0 | 1793 | 0.2 |
| Polymyalgia rheumatica | 726.30 | 717.9 | 725 | M35.3 | 36312 | 3.4 |
| Polymyositis/dermatomyositis | 710.00, 710.01 | 716.0, 716.1 | 710D, 710E | M33 | 3370 | 0.3 |
| Primary biliary cirrhosis | none | none | 571G | K74.3 | 5257 | 0.5 |
| Psoriasis | 706 | 696 | 696 | L40 | 173875 | 16.5 |
| Reiter’s disease | 138.1 | 136.03 | 099D, 711B | M02.3 | 1646 | 0.2 |
| Rheumatic fever | 400-401 | 390-392, excluding 392.9 | 390-391 | I00, I01 | 4202 | 0.4 |
| Rheumatoid arthritis | 722.0, excluding 722.1 and 722.2 | 712.0,712.1, 712.3 | 714, excluding 714E, 714X | M05, M06, M08.0, M08.2 | 130508 | 12.4 |
| Sarcoidosis | 138.0 | 135 | 135 | D86 | 26243 | 2.5 |
| Sjögren’s syndrome | 374.06 | 734.9 | 710C | M35.0 | 13686 | 1.3 |
| Systemic lupus erythematosus | 456.2 | 734.1 | 710A | M32 | 10993 | 1.0 |
| Systemic sclerosis | 710.0(not 710.00, 710.01,710.07) | 734.0 | 710B | M34 | 4172 | 0.4 |
| Takayasus disease | 456.3 | none | 446H | M31.4 | 489 | 0.0 |
| Thrombotic thrombocytopenic purpura | none | none | 446G | M31.1 | 537 | 0.1 |
| Ulcerative colitis | 572.20, 572.21 | 563.10, 569.02 | 556 | K51 | 78567 | 7.4 |
| Wegener’s granulomatosis | none | 446.3 | 446E | M31.3 | 16216 | 1.5 |
| All |  |  |  |  | 1055226 | 100.0 |

| **Supplementary Table S2. Study population and number of events of pancreas cancer, 1964-2018.** | | | | | | | | |
| --- | --- | --- | --- | --- | --- | --- | --- | --- |
|  | Population | | | Events | | Rate per 100000 person years | | |
|  | No. | | % | No. | % | Rate* | 95% CI | |
| **Age at diagnosis (years)** | | 16494957 |  | 60086 |  |  |  |  |
| <50 | |  |  | 2119 | 3.5 |  |  |  |
| 50-59 | |  |  | 6574 | 10.9 |  |  |  |
| 60-69 | |  |  | 15964 | 26.6 |  |  |  |
| 70-79 | |  |  | 22128 | 36.8 |  |  |  |
| 80+ | |  |  | 13301 | 22.1 |  |  |  |
| Gender | |  |  |  |  |  |  |  |
| Males | | 8382433 | 50.8 | 30426 | 50.6 | 7.37 | 7.29 | 7.46 |
| Females | | 8112524 | 49.2 | 29660 | 49.4 | 5.83 | 5.77 | 5.9 |
| **Period (years)** | | |  |  |  |  |  |  |
| 1964-73 | | |  | 9858 | 16.4 | 8.18 | 8.02 | 8.34 |
| 1974-83 | | |  | 12064 | 20.1 | 7.99 | 7.85 | 8.13 |
| 1984-93 | | |  | 12055 | 20.1 | 7.12 | 7 | 7.25 |
| 1994-03 | |  |  | 9380 | 15.6 | 5.37 | 5.26 | 5.47 |
| 2004-13 | | |  | 9842 | 16.4 | 5.26 | 5.16 | 5.37 |
| 2014-18 | |  |  | 6887 | 11.5 | 7.17 | 7.01 | 7.35 |
| **Region of residence** | | |  |  |  |  |  |  |
| Large cities | | 11066451 | 67.1 | 41986 | 69.9 | 9.01 | 8.93 | 9.1 |
| Southern Sweden | | 3944094 | 23.9 | 13191 | 22 | 4.08 | 4.01 | 4.15 |
| Northern Sweden | | 1484412 | 9.0 | 4909 | 8.2 | 4.04 | 3.92 | 4.15 |
| **Education** | |  |  |  |  |  |  |  |
| Low | | 8767297 | 53.2 | 44599 | 74.2 | 7.59 | 7.52 | 7.66 |
| Media | | 2209171 | 13.4 | 7551 | 12.6 | 5.28 | 5.16 | 5.4 |
| High | | 5518489 | 33.5 | 7936 | 13.2 | 5.37 | 5.26 | 5.49 |
| **Country of origin** | |  |  |  |  |  |  |  |
| Born in Sweden | | 13432893 | 81.4 | 55360 | 92.1 | 6.59 | 6.53 | 6.64 |
| Born in another place | | 3062064 | 18.6 | 4726 | 7.9 | 6.18 | 6.01 | 6.36 |
| **Diagnosis of obesity** | |  |  |  |  |  |  |  |
| Yes | | 185469 | 1.1 | 257 | 0.4 | 6.01 | 5.32 | 6.79 |
| No | | 16309488 | 98.9 | 59829 | 99.6 | 6.56 | 6.51 | 6.61 |
| **Diagnosis of alcoholism** | |  |  |  |  |  |  |  |
| Yes | | 413600 | 2.5 | 1431 | 2.4 | 7.94 | 7.54 | 8.37 |
| No | | 16081357 | 97.5 | 58655 | 97.6 | 6.53 | 6.47 | 6.58 |
| **Diagnosis of chronic obstructive pulmonary disease** | |  |  |  |  |  |  |  |
| Yes | | 864664 | 5.2 | 1706 | 2.8 | 3.13 | 2.98 | 3.28 |
| No | | 15630293 | 94.8 | 58380 | 97.2 | 6.77 | 6.71 | 6.82 |
| **Diagnosis of diabetes** | |  |  |  |  |  |  |  |
| Yes | | 470847 | 2.9 | 4519 | 7.5 | 7.93 | 7.7 | 8.16 |
| No | | 16024110 | 97.1 | 55567 | 92.5 | 6.47 | 6.41 | 6.52 |
| All | 16494957 | | 100.0 | 60086 | 100 | 6.56 | 6.50 | 6.61 |
| *: Adjusted for world standardized population. | | | | | | | | |

| **Supplementary Table S3**. **Subsequent incidence risks of pancreas cancer of patients with autoimmune disease in men, 1964-2018.** | | | | | | | | | | | | | | | |  |  |  |  | |
| --- | --- | --- | --- | --- | --- | --- | --- | --- | --- | --- | --- | --- | --- | --- | --- | --- | --- | --- | --- | --- |
|  | <1 | | | |  | 1-5 | | | |  | 5-9 | | | |  | ≥ 10 | | | |  |
| Comorbidities | O | SIR | 95% CI | |  | O | SIR | 95% CI | |  | O | SIR | 95% CI | |  | O | SIR | 95% CI | |  |
| Addison disease | 0 |  |  |  |  | 2 | 2.53 | 0.24 | 9.31 |  | 0 |  |  |  |  | 2 | 1.50 | 0.14 | 5.53 | |
| Ankylosing spondylitis | 1 | 1.39 | 0.00 | 7.96 |  | 6 | 1.15 | 0.41 | 2.51 |  | 5 | 0.85 | 0.27 | 2.00 |  | 22 | 1.17 | 0.73 | 1.77 | |
| Celiac disease | 12 | **17.91** | **9.21** | **31.38** |  | 8 | 1.67 | 0.71 | 3.31 |  | 8 | 1.63 | 0.69 | 3.22 |  | 5 | 0.75 | 0.24 | 1.76 | |
| Chronic rheumatic heart disease | 3 | 1.11 | 0.21 | 3.29 |  | 6 | 0.45 | 0.16 | 0.98 |  | 11 | 0.90 | 0.45 | 1.62 |  | 26 | 1.34 | 0.87 | 1.96 | |
| Crohn disease | 8 | **7.02** | **3.00** | **13.90** |  | 12 | 1.56 | 0.80 | 2.74 |  | 10 | 1.21 | 0.58 | 2.23 |  | 26 | 0.95 | 0.62 | 1.39 | |
| Dermatitis Herpetiformis | 0 |  |  |  |  | 2 | 1.20 | 0.11 | 4.43 |  | 2 | 1.16 | 0.11 | 4.28 |  | 4 | 1.34 | 0.35 | 3.47 | |
| Diabetes mellitus type I | 108 | **13.19** | **10.82** | **15.92** |  | 72 | 1.26 | 0.98 | 1.59 |  | 56 | 1.07 | 0.81 | 1.40 |  | 47 | 1.12 | 0.83 | 1.50 | |
| Discoid lupus erythematosus | 0 |  |  |  |  | 3 | 3.19 | 0.60 | 9.45 |  | 2 | 2.08 | 0.20 | 7.66 |  | 2 | 1.68 | 0.16 | 6.18 | |
| Giant-cell arteritis | 1 | 0.57 | 0.00 | 3.29 |  | 9 | 0.89 | 0.40 | 1.69 |  | 6 | 0.81 | 0.29 | 1.77 |  | 8 | 1.61 | 0.69 | 3.19 | |
| Glomerluar nephritis chronic | 4 | 2.33 | 0.60 | 6.01 |  | 4 | 0.54 | 0.14 | 1.39 |  | 7 | 1.05 | 0.41 | 2.17 |  | 11 | 0.78 | 0.39 | 1.40 | |
| Glomerular nephritis acute | 2 | 6.67 | 0.63 | 24.52 |  | 0 | 0.00 | 0.62 | 2.55 |  | 1 | 0.63 | 0.00 | 3.61 |  | 5 | 0.58 | 0.18 | 1.36 | |
| Grave disease | 4 | 3.20 | 0.83 | 8.27 |  | 10 | 1.17 | 0.56 | 2.17 |  | 8 | 0.95 | 0.40 | 1.87 |  | 30 | **1.53** | **1.03** | **2.18** | |
| Guillain-Barre Syndrome | 2 | 5.56 | 0.52 | 20.43 |  | 2 | 0.86 | 0.08 | 3.16 |  | 3 | 1.30 | 0.24 | 3.84 |  | 5 | 1.04 | 0.33 | 2.45 | |
| Hashimoto thyroiditis | 5 | **4.46** | **1.41** | **10.50** |  | 11 | 1.72 | 0.85 | 3.08 |  | 3 | 0.60 | 0.11 | 1.77 |  | 7 | 1.24 | 0.49 | 2.57 | |
| Immune thrombocytopenic purpura | 1 | 1.28 | 0.00 | 7.35 |  | 8 | 1.91 | 0.82 | 3.78 |  | 1 | 0.33 | 0.00 | 1.89 |  | 10 | **2.36** | **1.12** | **4.35** | |
| Lupoid hepatitis | 3 | **9.68** | **1.82** | **28.65** |  | 3 | 1.75 | 0.33 | 5.19 |  | 1 | 0.56 | 0.00 | 3.20 |  | 10 | 1.47 | 0.70 | 2.71 | |
| Multiple sclerosis | 2 | 2.70 | 0.25 | 9.94 |  | 4 | 0.78 | 0.20 | 2.00 |  | 4 | 0.67 | 0.17 | 1.73 |  | 7 | 0.50 | 0.20 | 1.04 | |
| Myasthenia gravis | 0 |  |  |  |  | 1 | 0.37 | 0.00 | 2.11 |  | 1 | 0.43 | 0.00 | 2.48 |  | 1 | 0.34 | 0.00 | 1.92 | |
| Pemphigoid | 0 |  |  |  |  | 10 | **2.15** | **1.02** | **3.97** |  | 3 | 1.47 | 0.28 | 4.35 |  | 0 |  |  |  | |
| Pernicious anemia | 11 | **5.79** | **2.87** | **10.39** |  | 14 | 1.25 | 0.68 | 2.10 |  | 15 | 1.69 | 0.94 | 2.79 |  | 15 | 1.45 | 0.81 | 2.41 | |
| Polymyalgia rheumatica | 8 | **2.62** | **1.12** | **5.19** |  | 20 | 1.12 | 0.68 | 1.73 |  | 10 | 0.76 | 0.36 | 1.41 |  | 5 | 0.51 | 0.16 | 1.21 | |
| Polymyositis/dermatomyositis | 1 | 4.35 | 0.00 | 24.92 |  | 1 | 0.80 | 0.00 | 4.59 |  | 2 | 1.94 | 0.18 | 7.14 |  | 2 | 1.52 | 0.14 | 5.57 | |
| Primary biliary cirrhosis | 2 | 7.69 | 0.73 | 28.29 |  | 0 |  |  |  |  | 0 |  |  |  |  | 0 |  |  |  | |
| Psoriasis | 4 | 0.57 | 0.15 | 1.48 |  | 55 | 1.06 | 0.80 | 1.39 |  | 58 | 1.17 | 0.89 | 1.51 |  | 74 | **1.27** | **1.00** | **1.59** | |
| Rheumatic fever | 0 |  |  |  |  | 6 | **3.51** | **1.26** | **7.69** |  | 2 | 1.00 | 0.09 | 3.66 |  | 17 | 1.51 | 0.88 | 2.42 | |
| Rheumatoid arthritis | 14 | **1.97** | **1.08** | **3.32** |  | 38 | 0.80 | 0.57 | 1.10 |  | 40 | 0.92 | 0.66 | 1.26 |  | 48 | 0.83 | 0.62 | 1.11 | |
| Sarcoidosis | 10 | **10.64** | **5.07** | **19.64** |  | 6 | 0.96 | 0.34 | 2.09 |  | 5 | 0.76 | 0.24 | 1.79 |  | 26 | 1.20 | 0.79 | 1.77 | |
| Sjögren syndrome | 0 |  |  |  |  | 0 |  |  |  |  | 2 | 2.11 | 0.20 | 7.74 |  | 1 | 1.30 | 0.00 | 7.44 | |
| Systemic lupus erythematosus | 1 | 3.45 | 0.00 | 19.77 |  | 4 | 2.30 | 0.60 | 5.94 |  | 0 |  |  |  |  | 5 | 2.07 | 0.65 | 4.88 | |
| Systemic sclerosis | 0 |  |  |  |  | 2 | 2.13 | 0.20 | 7.82 |  | 0 |  |  |  |  | 4 | 3.42 | 0.89 | 8.84 | |
| Ulcerative colitis | 6 | 2.28 | 0.82 | 5.00 |  | 19 | 1.02 | 0.61 | 1.59 |  | 24 | 1.17 | 0.75 | 1.75 |  | 78 | **1.45** | **1.15** | **1.81** | |
| Wegener granulomatosis | 4 | 2.23 | 0.58 | 5.78 |  | 9 | 0.79 | 0.36 | 1.50 |  | 9 | 0.96 | 0.44 | 1.83 |  | 9 | 0.95 | 0.43 | 1.81 | |
| All | 223 | **4.29** | **3.75** | **4.89** |  | 350 | 1.07 | 0.96 | 1.19 |  | 306 | 1.03 | 0.92 | 1.15 |  | 522 | **1.15** | **1.05** | **1.25** | |
| CI, confidence intervals; O, observed cases; SIR, standardized incidence ratio. | | | | | | | | | | | | | | | | | | | |  |
| Bold type: 95% confidence interval does not include 1.00. | | | | | | | | | | | | | | | | | | | |  |
| Adjusted for age, period, socioeconomic status, region of residence, and comorbidities. | | | | | | | | | | | | | | | | | | | |  |

| **Supplementary Table S4**. **Subsequent incidence risks of pancreas cancer of patients with autoimmune disease in women, 1964-2018.** | | | | | | | | | | | | | | | |  | |  |  |  |  | | |  | | |  | | |  |  |
| --- | --- | --- | --- | --- | --- | --- | --- | --- | --- | --- | --- | --- | --- | --- | --- | --- | --- | --- | --- | --- | --- | --- | --- | --- | --- | --- | --- | --- | --- | --- | --- |
|  | <1 | | | |  | 1-5 | | | |  | 5-9 | | | |  | | ≥10 | | | | | |  | | |  | | |  | | |
| Comorbidities | O | SIR | 95% CI | |  | O | SIR | 95% CI | |  | O | SIR | 95% CI | |  | | O | SIR | 95% CI | |  | | |  | | |  | | |  |  |
| Addison disease | 1 | 5.26 | 0.00 | 30.17 |  | 2 | 1.75 | 0.17 | 6.45 |  | 1 | 0.88 | 0.00 | 5.07 |  | | 2 | 0.88 | 0.08 | 3.25 |  | | |  | | |  | | |  |  |
| Ankylosing spondylitis | 0 |  |  |  |  | 3 | 1.52 | 0.29 | 4.49 |  | 0 |  |  |  |  | | 9 | 1.21 | 0.55 | 2.30 |  | | |  | | |  | | |  |  |
| Celiac disease | 9 | **11.25** | **5.10** | **21.45** |  | 17 | **2.73** | **1.59** | **4.39** |  | 6 | 0.84 | 0.30 | 1.84 |  | | 13 | 1.16 | 0.61 | 1.98 |  | | |  | | |  | | |  |  |
| Chronic rheumatic heart disease | 3 | 1.04 | 0.20 | 3.07 |  | 16 | 1.09 | 0.62 | 1.77 |  | 10 | 0.78 | 0.37 | 1.45 |  | | 21 | 0.98 | 0.61 | 1.51 |  | | |  | | |  | | |  |  |
| Crohn disease | 4 | 3.57 | 0.93 | 9.23 |  | 14 | 1.77 | 0.97 | 2.98 |  | 12 | 1.36 | 0.70 | 2.39 |  | | 28 | 0.95 | 0.63 | 1.38 |  | | |  | | |  | | |  |  |
| Dermatitis Herpetiformis | 1 | 8.33 | 0.00 | 47.77 |  | 2 | 2.22 | 0.21 | 8.17 |  | 1 | 0.98 | 0.00 | 5.62 |  | | 2 | 0.97 | 0.09 | 3.55 |  | | |  | | |  | | |  |  |
| Diabetes mellitus type I | 79 | **14.47** | **11.45** | **18.04** |  | 43 | 1.14 | 0.82 | 1.53 |  | 27 | 0.78 | 0.52 | 1.14 |  | | 23 | 0.80 | 0.51 | 1.21 |  | | |  | | |  | | |  |  |
| Discoid lupus erythematosus | 2 | 6.90 | 0.65 | 25.36 |  | 2 | 0.92 | 0.09 | 3.39 |  | 1 | 0.45 | 0.00 | 2.61 |  | | 8 | **2.57** | **1.10** | **5.09** |  | | |  | | |  | | |  |  |
| Giant-cell arteritis | 4 | 1.26 | 0.33 | 3.25 |  | 25 | 1.23 | 0.79 | 1.82 |  | 14 | 0.84 | 0.46 | 1.42 |  | | 14 | 1.19 | 0.65 | 2.00 |  | | |  | | |  | | |  |  |
| Glomerluar nephritis chronic | 1 | 1.15 | 0.00 | 6.59 |  | 6 | 1.69 | 0.61 | 3.69 |  | 2 | 0.65 | 0.06 | 2.38 |  | | 5 | 0.65 | 0.21 | 1.53 |  | | |  | | |  | | |  |  |
| Glomerular nephritis acute | 0 |  |  |  |  | 0 |  |  |  |  | 0 |  |  |  |  | | 4 | 0.80 | 0.21 | 2.06 |  | | |  | | |  | | |  |  |
| Grave disease | 14 | **3.08** | **1.68** | **5.18** |  | 30 | 0.93 | 0.62 | 1.32 |  | 47 | **1.41** | **1.03** | **1.87** |  | | 94 | 1.06 | 0.86 | 1.30 |  | | |  | | |  | | |  |  |
| Guillain-Barre Syndrome | 0 |  |  |  |  | 2 | 1.37 | 0.13 | 5.04 |  | 0 |  |  |  |  | | 2 | 0.59 | 0.06 | 2.18 |  | | |  | | |  | | |  |  |
| Hashimoto thyroiditis | 10 | **2.62** | **1.25** | **4.83** |  | 24 | 0.97 | 0.62 | 1.45 |  | 18 | 0.85 | 0.50 | 1.34 |  | | 26 | 0.90 | 0.59 | 1.32 |  | | |  | | |  | | |  |  |
| Immune thrombocytopenic purpura | 4 | **5.80** | **1.51** | **14.99** |  | 3 | 0.73 | 0.14 | 2.15 |  | 1 | 0.28 | 0.00 | 1.61 |  | | 12 | 1.73 | 0.89 | 3.04 |  | | |  | | |  | | |  |  |
| Lupoid hepatitis | 4 | **8.00** | **2.08** | **20.69** |  | 4 | 1.30 | 0.34 | 3.37 |  | 4 | 1.34 | 0.35 | 3.47 |  | | 10 | 1.19 | 0.57 | 2.20 |  | | |  | | |  | | |  |  |
| Multiple sclerosis | 3 | 2.97 | 0.56 | 8.79 |  | 3 | 0.38 | 0.07 | 1.13 |  | 5 | 0.48 | 0.15 | 1.13 |  | | 25 | 0.95 | 0.61 | 1.40 |  | | |  | | |  | | |  |  |
| Myasthenia gravis | 1 | 3.33 | 0.00 | 19.11 |  | 2 | 1.03 | 0.10 | 3.79 |  | 1 | 0.58 | 0.00 | 3.35 |  | | 7 | 1.88 | 0.75 | 3.90 |  | | |  | | |  | | |  |  |
| Pemphigoid | 3 | 2.78 | 0.52 | 8.22 |  | 3 | 0.64 | 0.12 | 1.91 |  | 2 | 0.81 | 0.08 | 2.98 |  | | 3 | 1.95 | 0.37 | 5.77 |  | | |  | | |  | | |  |  |
| Pernicious anemia | 4 | 2.05 | 0.53 | 5.30 |  | 20 | 1.62 | 0.99 | 2.51 |  | 14 | 1.41 | 0.77 | 2.38 |  | | 18 | 1.57 | 0.93 | 2.48 |  | | |  | | |  | | |  |  |
| Polymyalgia rheumatica | 9 | **1.89** | **0.86** | **3.60** |  | 37 | 1.25 | 0.88 | 1.73 |  | 25 | 1.09 | 0.71 | 1.62 |  | | 19 | 1.07 | 0.64 | 1.67 |  | | |  | | |  | | |  |  |
| Polymyositis/dermatomyositis | 2 | 8.00 | 0.75 | 29.42 |  | 5 | **3.47** | **1.10** | **8.17** |  | 2 | 1.53 | 0.14 | 5.61 |  | | 2 | 0.96 | 0.09 | 3.54 |  | | |  | | |  | | |  |  |
| Primary biliary cirrhosis | 1 | 1.49 | 0.00 | 8.56 |  | 4 | 1.27 | 0.33 | 3.29 |  | 0 |  |  |  |  | | 7 | 1.50 | 0.59 | 3.10 |  | | |  | | |  | | |  |  |
| Psoriasis | 22 | **3.06** | **1.92** | **4.65** |  | 69 | 1.27 | 0.99 | 1.61 |  | 77 | **1.42** | **1.12** | **1.77** |  | | 95 | **1.57** | **1.27** | **1.92** |  | | |  | | |  | | |  |  |
| Rheumatic fever | 0 |  |  |  |  | 3 | 3.19 | 0.60 | 9.45 |  | 1 | 1.02 | 0.00 | 5.85 |  | | 2 | 0.52 | 0.05 | 1.91 |  | | |  | | |  | | |  |  |
| Rheumatoid arthritis | 19 | 1.48 | 0.89 | 2.32 |  | 87 | 0.95 | 0.76 | 1.18 |  | 87 | 0.95 | 0.76 | 1.17 |  | | 156 | 1.03 | 0.87 | 1.20 |  | | |  | | |  | | |  |  |
| Sarcoidosis | 3 | 2.88 | 0.54 | 8.54 |  | 4 | 0.54 | 0.14 | 1.38 |  | 18 | **2.16** | **1.28** | **3.42** |  | | 18 | 0.74 | 0.44 | 1.17 |  | | |  | | |  | | |  |  |
| Sjögren syndrome | 1 | 0.76 | 0.00 | 4.34 |  | 9 | 0.93 | 0.42 | 1.78 |  | 19 | **2.09** | **1.25** | **3.27** |  | | 7 | 0.79 | 0.31 | 1.64 |  | | |  | | |  | | |  |  |
| Systemic lupus erythematosus | 2 | 2.74 | 0.26 | 10.08 |  | 11 | **2.25** | **1.12** | **4.05** |  | 7 | 1.35 | 0.53 | 2.79 |  | | 22 | **1.83** | **1.15** | **2.78** |  | | |  | | |  | | |  |  |
| Systemic sclerosis | 1 | 2.78 | 0.00 | 15.92 |  | 1 | 0.44 | 0.00 | 2.54 |  | 1 | 0.44 | 0.00 | 2.54 |  | | 7 | 1.82 | 0.72 | 3.77 |  | | |  | | |  | | |  |  |
| Ulcerative colitis | 7 | **3.29** | **1.30** | **6.81** |  | 13 | 0.85 | 0.45 | 1.45 |  | 20 | 1.19 | 0.72 | 1.84 |  | | 54 | 1.24 | 0.93 | 1.62 |  | | |  | | |  | | |  |  |
| Wegener granulomatosis | 8 | **2.70** | **1.15** | **5.35** |  | 23 | 1.07 | 0.68 | 1.61 |  | 21 | 0.99 | 0.61 | 1.52 |  | | 26 | 1.03 | 0.67 | 1.51 |  | | |  | | |  | | |  |  |
| All | 226 | **3.43** | **3.00** | **3.91** |  | 491 | **1.12** | **1.02** | **1.22** |  | 447 | 1.06 | 0.97 | 1.17 |  | | 744 | **1.10** | **1.02** | **1.18** |  | | |  | | |  | | |  |  |
| CI, confidence intervals; O, observed cases; SIR, standardized incidence ratio. | | | | | | | | | | | | | | | | | | | | | |  | | |  | | |  | | |  |
| Bold type: 95% confidence interval does not include 1.00. | | | | | | | | | | | | | | | | | | | | | |  | | |  | | |  | | |  |
| Adjusted for age, period, socioeconomic status, region of residence, and comorbidities. | | | | | | | | | | | | | | | | | | | | | |  | | |  | | |  | | |  |

| **Supplementary Table S5. Subsequent mortality risk of pancreas cancer without autoimmune pancreatitis, 1964-2018.** | | | | | | | | | | | | | | | | | | |  | |  | | |  | | | | | | |  | |  | | | | |  | | |  | | |  |  |  |  |  |  |
| --- | --- | --- | --- | --- | --- | --- | --- | --- | --- | --- | --- | --- | --- | --- | --- | --- | --- | --- | --- | --- | --- | --- | --- | --- | --- | --- | --- | --- | --- | --- | --- | --- | --- | --- | --- | --- | --- | --- | --- | --- | --- | --- | --- | --- | --- | --- | --- | --- | --- |
|  | <1 | | | | |  | | 1-5 | | | | |  | | 5-9 | | | | | | | | | |  | | | ≥ 10 | | | | | | | | | | | | | | | |  |  |  |  |  |  |
| Comorbidities | O | SMR | 95% CI | |  | | O | | SMR | 95% CI | |  | | O | | SMR | 95% CI | | | | | |  | | | | O | | | SIR | | | | | 95% CI | | | | | |  |  |  |  |  |  |  |  |  |
| Addison disease | 3 | **7.32** | **1.38** | **21.66** |  | | 5 | | 2.03 | 0.64 | 4.78 |  | | 1 | | 0.40 | 0.00 | | | 2.28 | |  | | | | 4 | | | 0.84 | | | | | 0.22 | | | 2.16 | | |  |  |  |  |  |  |  |  |  |  |
| Amyotrophic lateral sclerosis | 4 | 1.12 | 0.29 | 2.90 |  | | 9 | | 1.26 | 0.57 | 2.41 |  | | 2 | | 0.66 | 0.06 | | | 2.41 | |  | | | | 4 | | | 0.98 | | | | | 0.25 | | | 2.53 | | |  |  |  |  |  |  |  |  |  |  |
| Ankylosing spondylitis | 2 | 1.74 | 0.16 | 6.40 |  | | 11 | | 1.33 | 0.66 | 2.39 |  | | 5 | | 0.54 | 0.17 | | | 1.27 | |  | | | | 27 | | | 0.85 | | | | | 0.56 | | | 1.24 | | |  |  |  |  |  |  |  |  |  |  |
| Celiac disease | 10 | **5.52** | **2.63** | **10.20** |  | | 36 | | **2.63** | **1.84** | **3.65** |  | | 22 | | 1.44 | 0.90 | | | 2.18 | |  | | | | 22 | | | 0.94 | | | | | 0.59 | | | 1.43 | | |  |  |  |  |  |  |  |  |  |  |
| Chronic rheumatic heart disease | 4 | 0.60 | 0.16 | 1.55 |  | | 24 | | 0.71 | 0.45 | 1.05 |  | | 24 | | 0.77 | 0.49 | | | 1.15 | |  | | | | 66 | | | 1.15 | | | | | 0.89 | | | 1.46 | | |  |  |  |  |  |  |  |  |  |  |
| Crohn disease | 8 | **2.90** | **1.24** | **5.74** |  | | 31 | | **1.62** | **1.10** | **2.30** |  | | 28 | | 1.32 | 0.88 | | | 1.91 | |  | | | | 75 | | | 1.08 | | | | | 0.85 | | | 1.36 | | |  |  |  |  |  |  |  |  |  |  |
| Dermatitis Herpetiformis | 1 | 2.33 | 0.00 | 13.33 |  | | 4 | | 1.20 | 0.31 | 3.11 |  | | 3 | | 0.82 | 0.15 | | | 2.43 | |  | | | | 8 | | | 1.13 | | | | | 0.48 | | | 2.23 | | |  |  |  |  |  |  |  |  |  |  |
| Diabetes mellitus type I | 151 | **8.23** | **6.97** | **9.66** |  | | 315 | | **2.55** | **2.28** | **2.85** |  | | 133 | | **1.22** | **1.02** | | | **1.45** | |  | | | | 78 | | | 0.92 | | | | | 0.72 | | | 1.14 | | |  |  |  |  |  |  |  |  |  |  |
| Discoid lupus erythematosus | 0 |  |  |  |  | | 5 | | 1.22 | 0.38 | 2.87 |  | | 4 | | 0.96 | 0.25 | | | 2.47 | |  | | | | 11 | | | 1.90 | | | | | 0.94 | | | 3.41 | | |  |  |  |  |  |  |  |  |  |  |
| Giant-cell arteritis | 5 | 0.70 | 0.22 | 1.65 |  | | 72 | | **1.56** | **1.22** | **1.96** |  | | 26 | | 0.64 | 0.42 | | | 0.94 | |  | | | | 48 | | | **1.47** | | | | | **1.08** | | | **1.95** | | |  |  |  |  |  |  |  |  |  |  |
| Glomerluar nephritis chronic | 4 | 1.34 | 0.35 | 3.46 |  | | 9 | | 0.70 | 0.32 | 1.34 |  | | 13 | | 1.14 | 0.60 | | | 1.95 | |  | | | | 20 | | | 0.79 | | | | | 0.48 | | | 1.22 | | |  |  |  |  |  |  |  |  |  |  |
| Glomerular nephritis acute | 2 | 3.45 | 0.33 | 12.68 |  | | 2 | | 0.64 | 0.06 | 2.36 |  | | 2 | | 0.60 | 0.06 | | | 2.20 | |  | | | | 13 | | | 0.77 | | | | | 0.41 | | | 1.32 | | |  |  |  |  |  |  |  |  |  |  |
| Grave disease | 16 | **2.26** | **1.29** | **3.68** |  | | 59 | | 1.16 | 0.89 | 1.50 |  | | 62 | | 1.18 | 0.90 | | | 1.51 | |  | | | | 178 | | | **1.24** | | | | | **1.06** | | | **1.43** | | |  |  |  |  |  |  |  |  |  |  |
| Guillain-Barre Syndrome | 2 | 2.86 | 0.27 | 10.51 |  | | 3 | | 0.65 | 0.12 | 1.91 |  | | 4 | | 0.82 | 0.21 | | | 2.12 | |  | | | | 10 | | | 0.90 | | | | | 0.43 | | | 1.66 | | |  |  |  |  |  |  |  |  |  |  |
| Hashimoto thyroiditis | 13 | **2.04** | **1.08** | **3.50** |  | | 45 | | 1.11 | 0.81 | 1.49 |  | | 42 | | 1.22 | 0.88 | | | 1.65 | |  | | | | 50 | | | 1.04 | | | | | 0.77 | | | 1.37 | | |  |  |  |  |  |  |  |  |  |  |
| Immune thrombocytopenic purpura | 9 | **4.57** | **2.07** | **8.71** |  | | 14 | | 1.27 | 0.69 | 2.14 |  | | 5 | | 0.57 | 0.18 | | | 1.35 | |  | | | | 28 | | | **1.84** | | | | | **1.22** | | | **2.67** | | |  |  |  |  |  |  |  |  |  |  |
| Lupoid hepatitis | 5 | **5.32** | **1.68** | **12.51** |  | | 9 | | 1.63 | 0.74 | 3.11 |  | | 7 | | 1.27 | 0.50 | | | 2.63 | |  | | | | 21 | | | 1.11 | | | | | 0.69 | | | 1.70 | | |  |  |  |  |  |  |  |  |  |  |
| Multiple sclerosis | 4 | 2.06 | 0.54 | 5.33 |  | | 12 | | 0.84 | 0.43 | 1.46 |  | | 13 | | 0.71 | 0.38 | | | 1.21 | |  | | | | 47 | | | 0.96 | | | | | 0.70 | | | 1.27 | | |  |  |  |  |  |  |  |  |  |  |
| Myasthenia gravis | 0 |  |  |  |  | | 5 | | 0.80 | 0.25 | 1.88 |  | | 7 | | 1.25 | 0.49 | | | 2.58 | |  | | | | 11 | | | 1.16 | | | | | 0.58 | | | 2.08 | | |  |  |  |  |  |  |  |  |  |  |
| Pemphigoid | 3 | 0.79 | 0.15 | 2.32 |  | | 23 | | 1.44 | 0.91 | 2.16 |  | | 9 | | 1.17 | 0.53 | | | 2.24 | |  | | | | 2 | | | 0.46 | | | | | 0.04 | | | 1.69 | | |  |  |  |  |  |  |  |  |  |  |
| Pernicious anemia | 13 | **2.86** | **1.52** | **4.90** |  | | 37 | | 1.30 | 0.92 | 1.80 |  | | 32 | | 1.33 | 0.91 | | | 1.88 | |  | | | | 57 | | | **1.71** | | | | | **1.30** | | | **2.22** | | |  |  |  |  |  |  |  |  |  |  |
| Polymyalgia rheumatica | 17 | 1.48 | 0.86 | 2.38 |  | | 86 | | 1.18 | 0.94 | 1.46 |  | | 67 | | 1.10 | 0.85 | | | 1.40 | |  | | | | 55 | | | 1.05 | | | | | 0.79 | | | 1.37 | | |  |  |  |  |  |  |  |  |  |  |
| Polymyositis/dermatomyositis | 3 | 5.00 | 0.94 | 14.80 |  | | 5 | | 1.46 | 0.46 | 3.44 |  | | 6 | | 1.98 | 0.71 | | | 4.34 | |  | | | | 6 | | | 1.31 | | | | | 0.47 | | | 2.86 | | |  |  |  |  |  |  |  |  |  |  |
| Primary biliary cirrhosis | 3 | 2.68 | 0.50 | 7.93 |  | | 2 | | 0.39 | 0.04 | 1.45 |  | | 4 | | 0.82 | 0.21 | | | 2.13 | |  | | | | 11 | | | 1.49 | | | | | 0.74 | | | 2.67 | | |  |  |  |  |  |  |  |  |  |  |
| Psoriasis | 13 | 0.75 | 0.40 | 1.29 |  | | 148 | | 1.14 | 0.96 | 1.33 |  | | 149 | | 1.14 | 0.96 | | | 1.34 | |  | | | | 207 | | | **1.34** | | | | | **1.16** | | | **1.53** | | |  |  |  |  |  |  |  |  |  |  |
| Reiter disease | 0 |  |  |  |  | | 2 | | 4.08 | 0.38 | 15.01 |  | | 0 | |  |  | | |  | |  | | | | 8 | | | 1.24 | | | | | 0.53 | | | 2.45 | | |  |  |  |  |  |  |  |  |  |  |
| Rheumatic fever | 1 | 2.13 | 0.00 | 12.20 |  | | 10 | | **3.41** | **1.63** | **6.30** |  | | 4 | | 1.20 | 0.31 | | | 3.11 | |  | | | | 25 | | | 1.32 | | | | | 0.85 | | | 1.94 | | |  |  |  |  |  |  |  |  |  |  |
| Rheumatoid arthritis | 25 | 1.03 | 0.66 | 1.52 |  | | 161 | | 0.93 | 0.79 | 1.09 |  | | 162 | | 0.93 | 0.79 | | | 1.08 | |  | | | | 310 | | | 1.06 | | | | | 0.95 | | | 1.19 | | |  |  |  |  |  |  |  |  |  |  |
| Sarcoidosis | 5 | 2.16 | 0.68 | 5.09 |  | | 15 | | 0.94 | 0.52 | 1.55 |  | | 22 | | 1.25 | 0.78 | | | 1.89 | |  | | | | 67 | | | 1.11 | | | | | 0.86 | | | 1.42 | | |  |  |  |  |  |  |  |  |  |  |
| Sjögren syndrome | 3 | 1.55 | 0.29 | 4.58 |  | | 12 | | 0.85 | 0.44 | 1.49 |  | | 18 | | 1.35 | 0.80 | | | 2.13 | |  | | | | 14 | | | 1.04 | | | | | 0.57 | | | 1.75 | | |  |  |  |  |  |  |  |  |  |  |
| Systemic lupus erythematosus | 2 | 1.68 | 0.16 | 6.18 |  | | 14 | | 1.80 | 0.98 | 3.02 |  | | 10 | | 1.23 | 0.59 | | | 2.27 | |  | | | | 37 | | | **2.02** | | | | | **1.42** | | | **2.78** | | |  |  |  |  |  |  |  |  |  |  |
| Systemic sclerosis | 2 | 3.13 | 0.29 | 11.49 |  | | 5 | | 1.28 | 0.40 | 3.01 |  | | 1 | | 0.26 | 0.00 | | | 1.49 | |  | | | | 16 | | | **2.42** | | | | | **1.38** | | | **3.95** | | |  |  |  |  |  |  |  |  |  |  |
| Ulcerative colitis | 10 | 1.69 | 0.81 | 3.13 |  | | 48 | | 1.14 | 0.84 | 1.51 |  | | 62 | | **1.32** | **1.01** | | | **1.69** | |  | | | | 157 | | | **1.28** | | | | | **1.09** | | | **1.49** | | |  |  |  |  |  |  |  |  |  |  |
| Wegener granulomatosis | 10 | 1.83 | 0.87 | 3.38 |  | | 36 | | 0.94 | 0.66 | 1.31 |  | | 31 | | 0.83 | 0.57 | | | 1.19 | |  | | | | 55 | | | 0.98 | | | | | 0.74 | | | 1.28 | | |  |  |  |  |  |  |  |  |  |  |
| All | 356 | **2.38** | **2.14** | **2.65** |  | | 1284 | | **1.32** | **1.25** | **1.39** |  | | 991 | | **1.06** | **1.00** | | | **1.13** | |  | | | | 1757 | | | **1.15** | | | | | **1.10** | | | **1.21** | | |  |  |  |  |  |  |  |  |  |  |
| Autoimmune diseases with fewer than 10 cases were excluded but included in total numbers. | | | | | | | | | | | | | | | | | | | | | | | | | | | | | | | | | | | |  | | |  | | |  | | | | |  | |  |
| Abbreviations: O = Observed cases; SMR = Standardized mortality ratio; CI = Confidence intervals. | | | | | | | | | | | | | | | | | |  |  | |  | | |  | | | | | |  | |  | | | | |  | | |  | | |  | | | | |  | |
